# Supplementary material for: 2′-Deoxyuridine-promoted infection in Pyricularia oryzae is counteracted by bacterial thymidine phosphorylase
Source: Appl Environ Microbiol. 2026 Jul 13;92(8):e00727-26. doi: 10.1128/aem.00727-26 (PMC13488324; doi:10.1128/aem.00727-26)
Supplement: Supplementary file 1 [file aem.00727-26-s0001.pdf]

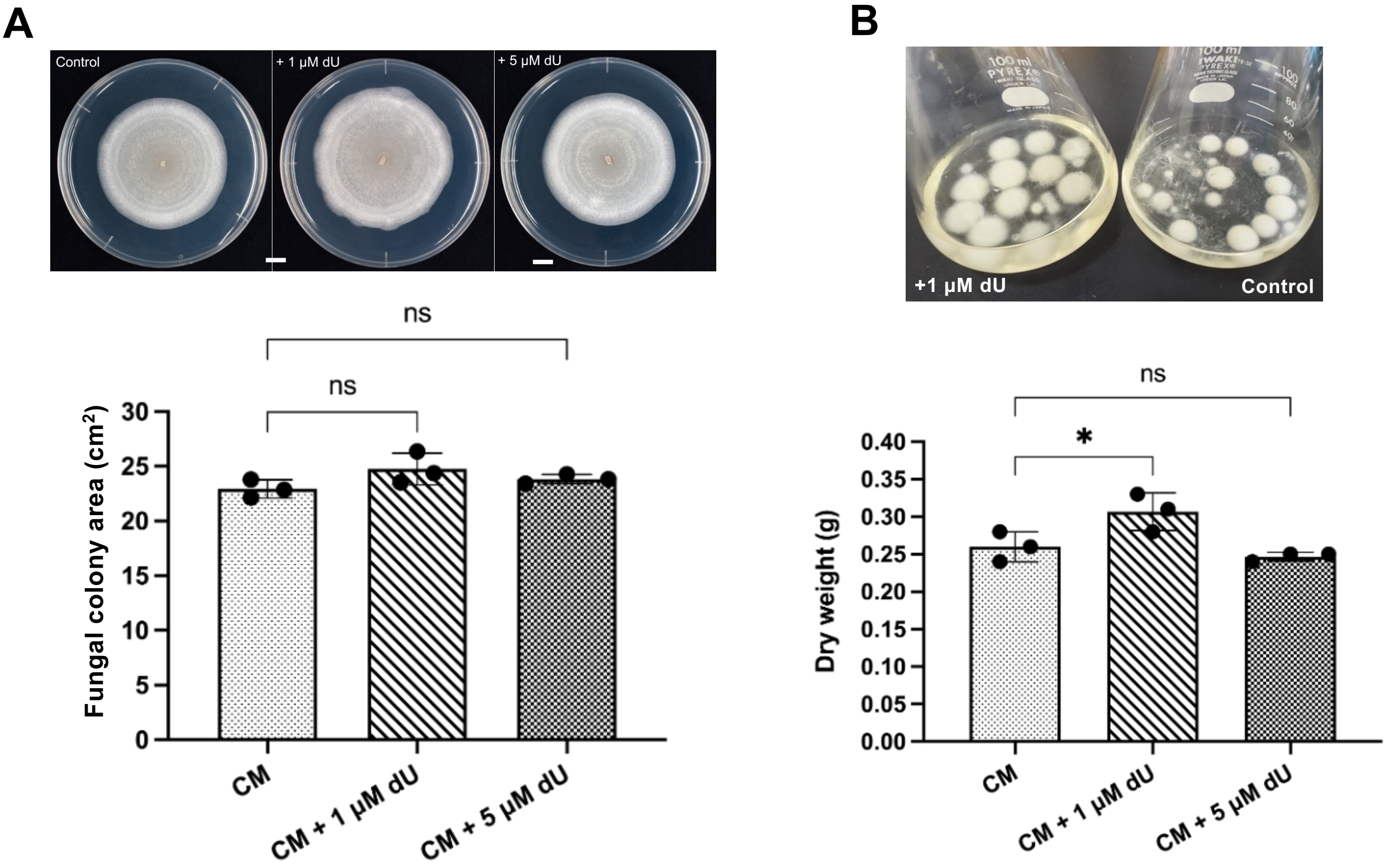

FIG S1 Effects of exogenous dU on vegetative growth of *Pyricularia oryzae*.
(A) Representative colonies (top) and colony area (bottom) of *P. oryzae* grown on complete medium (CM) with the indicated concentrations of dU for 11 days. (B) Representative liquid cultures (top) and dry weight of mycelia (bottom) grown in liquid CM with the indicated concentrations of dU for 6 days. Data represent means ± SD from three independent biological replicates. ns, not significant; * $P < 0.05$ (one-way ANOVA followed by Dunnett's multiple comparisons test).
