# Supplementary material for: 2′-Deoxyuridine-promoted infection in Pyricularia oryzae is counteracted by bacterial thymidine phosphorylase
Source: Appl Environ Microbiol. 2026 Jul 13;92(8):e00727-26. doi: 10.1128/aem.00727-26 (PMC13488324; doi:10.1128/aem.00727-26)
Supplement: Supplementary file 2 [file aem.00727-26-s0002.pdf]

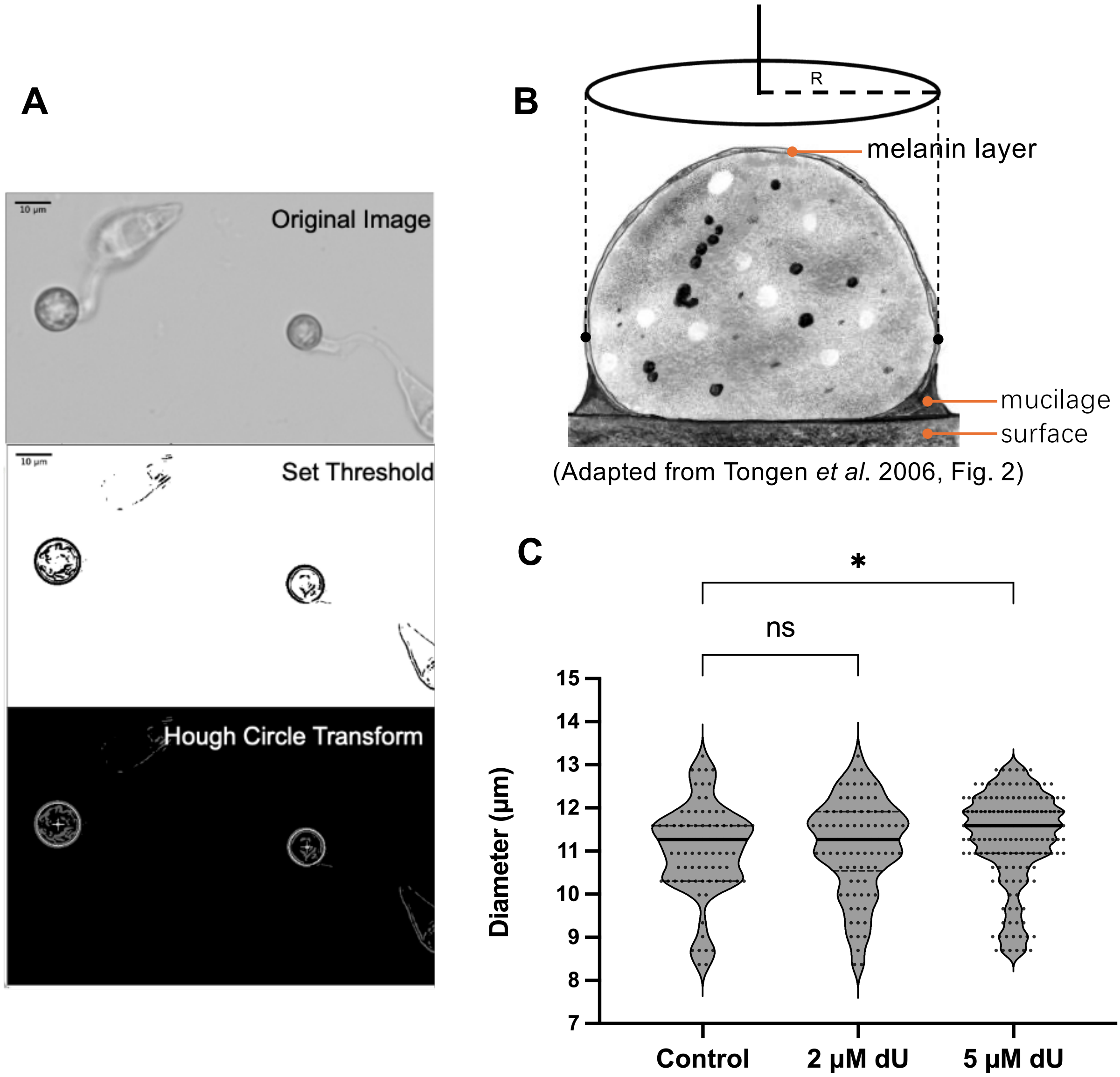

FIG S2 Effect of dU treatment on appressorium diameter of *Pyricularia oryzae*.
(A) Workflow for appressorium detection and diameter measurement using the Hough Circle Transform plugin in Fiji. (B) Schematic cross-section of an appressorium attached to the plant surface, illustrating the basis for diameter measurement. Adapted from Tongen et al. 2006, Fig. 2. (C) Violin plots showing appressorial diameter at 24 h in the control, 2 µM dU, and 5 µM dU groups. Individual data points are overlaid. The thick horizontal line indicates the median, and the dashed lines indicate the interquartile range (IQR). Statistical significance was determined using the Kruskal-Wallis test with Dunn's post-hoc test for multiple comparisons (* $P < 0.05$; ns, not significant), as the data did not pass the D'Agostino-Pearson normality test ($P < 0.05$).

Tongen A, Goriely A, Tabor M. 2006. Biomechanical model for appressorial design in *Magnaporthe grisea*. J Theor Biol 240:1–8. https://doi.org/10.1016/j.jtbi.2005.08.014
