# Supplementary material for: 2′-Deoxyuridine-promoted infection in Pyricularia oryzae is counteracted by bacterial thymidine phosphorylase
Source: Appl Environ Microbiol. 2026 Jul 13;92(8):e00727-26. doi: 10.1128/aem.00727-26 (PMC13488324; doi:10.1128/aem.00727-26)
Supplement: Supplementary file 3 [file aem.00727-26-s0003.pdf]

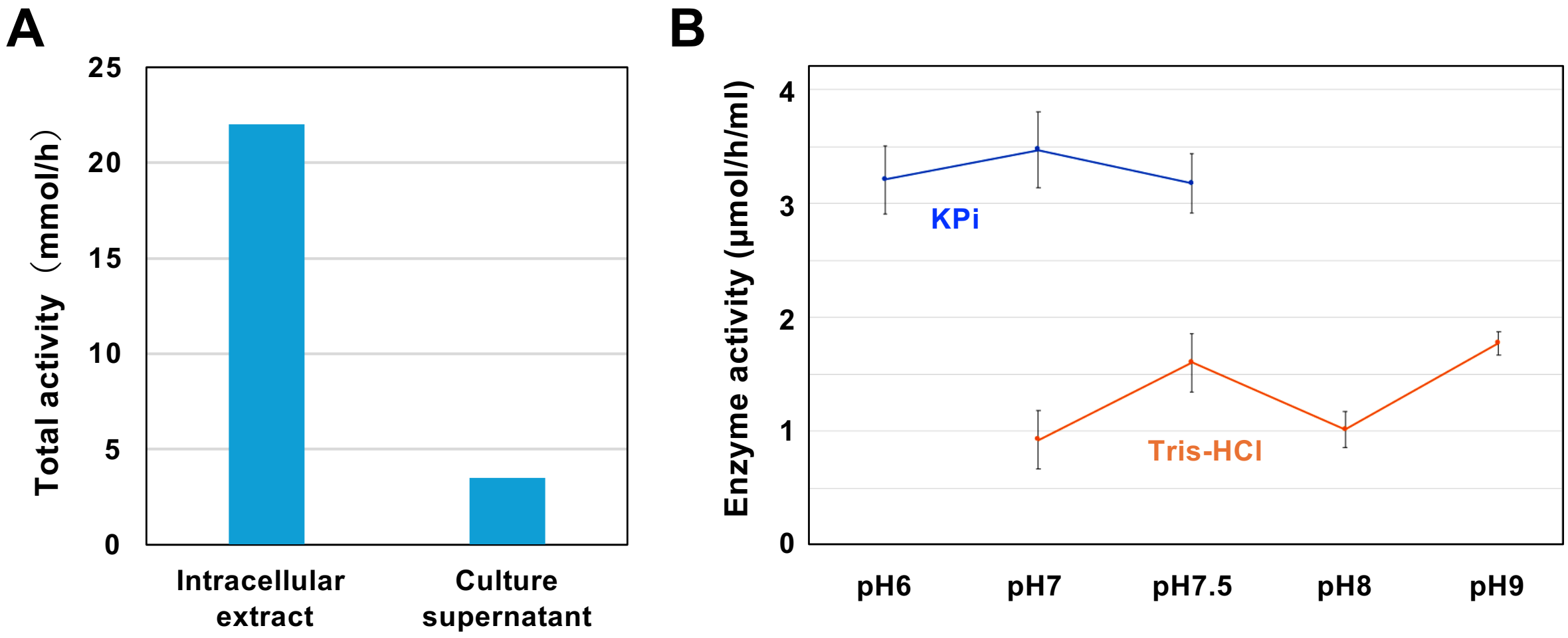

FIG S3 Intracellular localization and buffer dependence of dU-metabolizing activity in strain C3. (A) Total dU-metabolizing activity was compared between the crude intracellular extract and the culture supernatant of strain C3. (B) dU-metabolizing activity of the crude intracellular extract was examined in potassium phosphate (KPi) and Tris-HCl buffers at the indicated pH values.
