# Supplementary material for: 2′-Deoxyuridine-promoted infection in Pyricularia oryzae is counteracted by bacterial thymidine phosphorylase
Source: Appl Environ Microbiol. 2026 Jul 13;92(8):e00727-26. doi: 10.1128/aem.00727-26 (PMC13488324; doi:10.1128/aem.00727-26)
Supplement: Supplementary file 4 [file aem.00727-26-s0004.pdf]

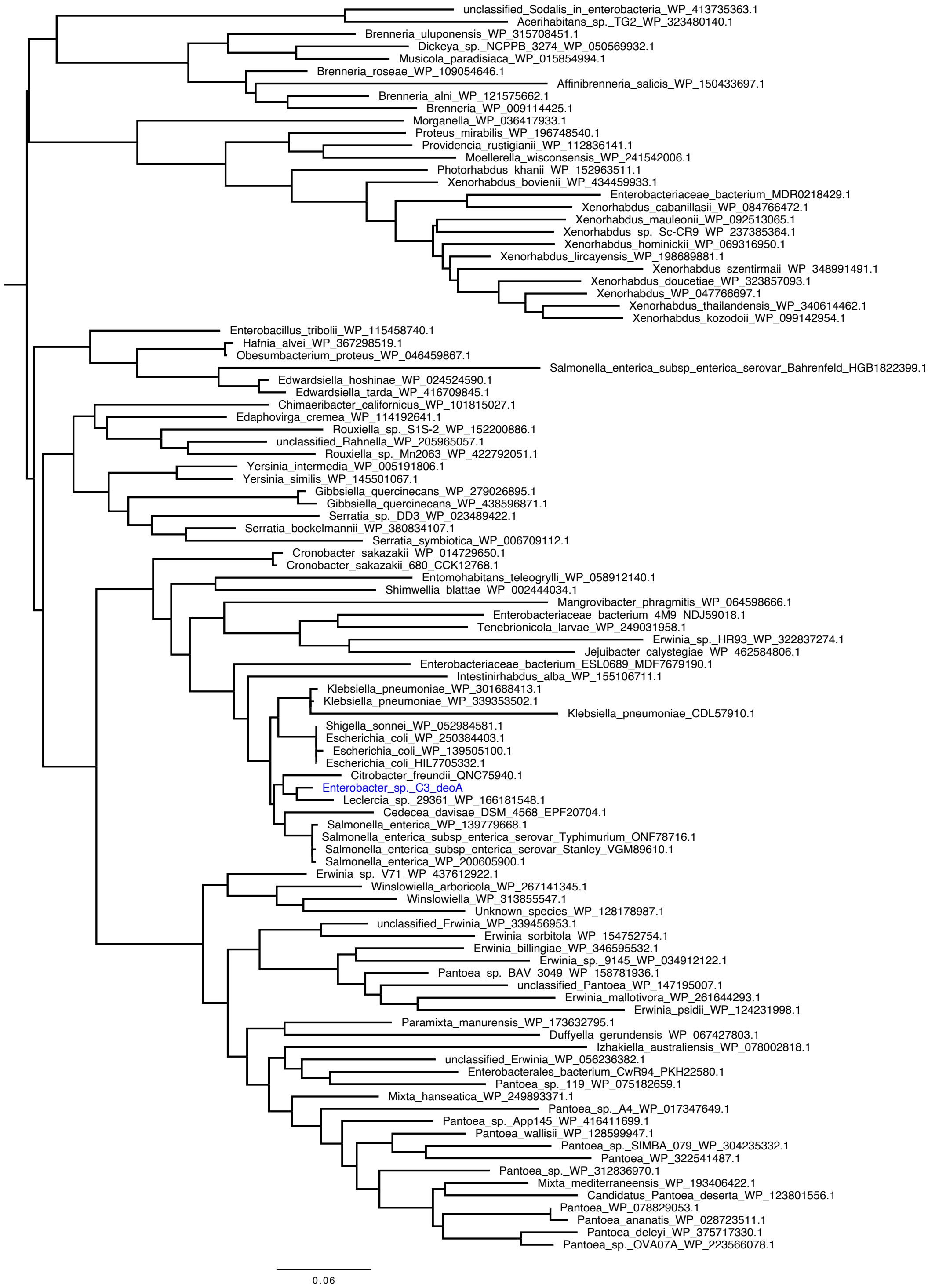

FIG S4 Maximum-likelihood phylogenetic tree of DeoA-like proteins from representative Enterobacterales. The tree was inferred from a trimmed amino acid alignment using IQ-TREE v3.0.1 under the Q.PFAM+I+R6 model. The DeoA sequence from *Enterobacter* sp. strain C3 is highlighted in blue. Taxon names are followed by protein accession numbers, and the sequences used in the analysis are listed in Table S2. The scale bar indicates amino acid substitutions per site.
