# Supplementary material for: 2′-Deoxyuridine-promoted infection in Pyricularia oryzae is counteracted by bacterial thymidine phosphorylase
Source: Appl Environ Microbiol. 2026 Jul 13;92(8):e00727-26. doi: 10.1128/aem.00727-26 (PMC13488324; doi:10.1128/aem.00727-26)
Supplement: Supplementary file 5 [file aem.00727-26-s0005.pdf]

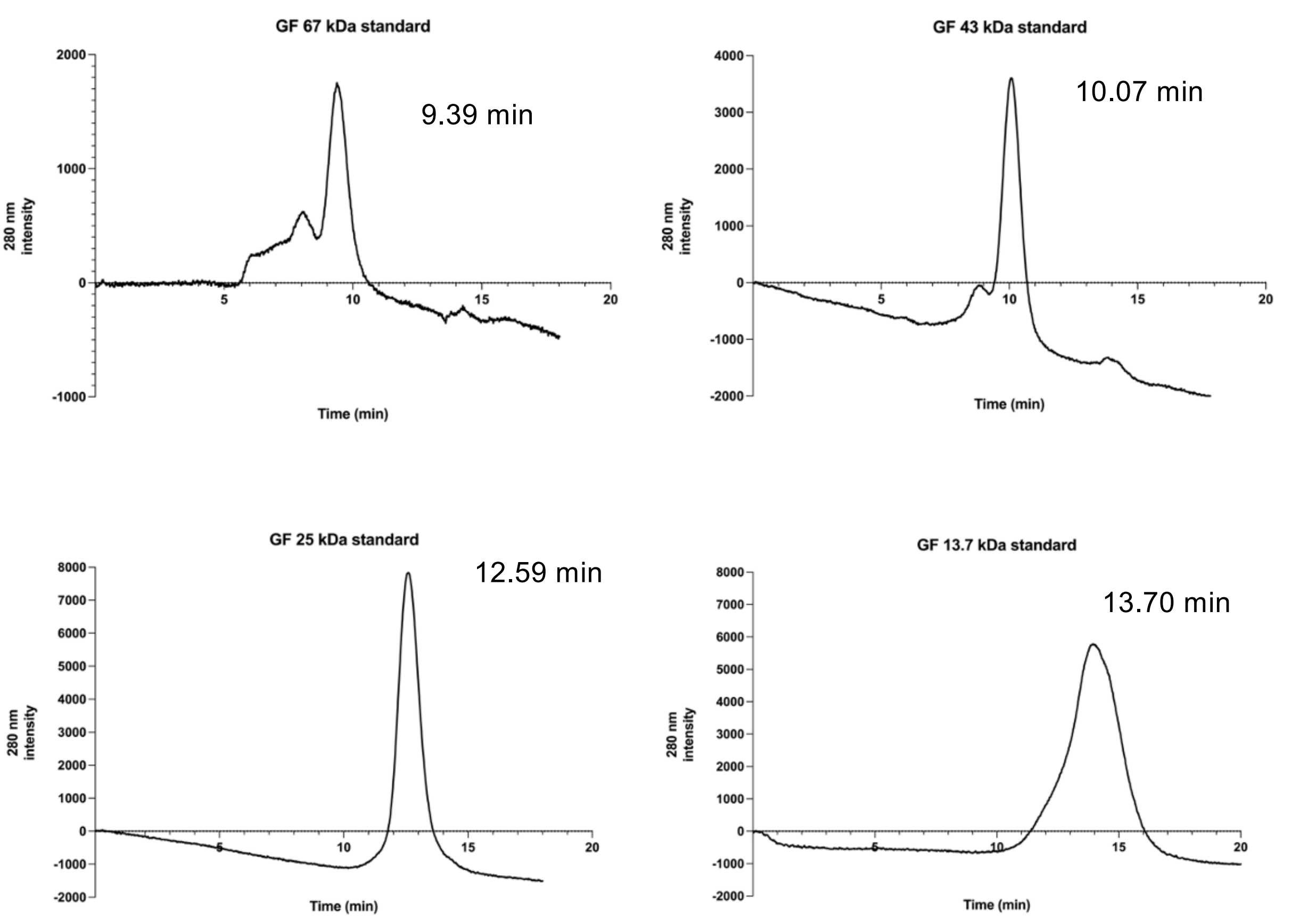

FIG S5 Calibration of the gel filtration column using standard protein markers.
Retention times (RT) for the protein standards were as follows: bovine serum albumin (67 kDa), 9.39 min; ovalbumin (43 kDa), 10.07 min; chymotrypsinogen A (25 kDa), 12.59 min; and ribonuclease A (13.7 kDa), 13.70 min.
