# Supplementary material for: 2′-Deoxyuridine-promoted infection in Pyricularia oryzae is counteracted by bacterial thymidine phosphorylase
Source: Appl Environ Microbiol. 2026 Jul 13;92(8):e00727-26. doi: 10.1128/aem.00727-26 (PMC13488324; doi:10.1128/aem.00727-26)
Supplement: Supplementary file 6 [file aem.00727-26-s0006.pdf]

Table S1a. Medium components of dUMM

Name of components	Concentration of components (g/ml)
2'-deoxyuridine	5.00×10^{-4}
Na_2HPO_4	5.70×10^{-4}
KH_2PO_4	3.60×10^{-4}
$MgSO_4 \cdot 7H_2O$	1.00×10^{-4}
$CaCl_2 \cdot 2H_2O$	1.47×10^{-5}
$(NH_4)_2SO_4$	5.00×10^{-4}
Metal Solution (Table S3b.)	2.00×10^{-3} ml/ml
Vitamin Solution (Table S3c.)	1.00×10^{-3} ml/ml

Table S1b. Medium components of Metal Solution

Name of components	Concentration of components (g/ml)
$FeSO_4 \cdot 7H_2O$	6.00×10^{-6}
$CoCl_2 \cdot 6H_2O$	3.80×10^{-7}
$MnCl_2 \cdot 4H_2O$	4.00×10^{-7}
$Na_2MoO_4 \cdot 2H_2O$	2.42×10^{-7}
$ZnCl_2$	1.40×10^{-7}
H_3BO_4	1.24×10^{-7}
$NiCl_2 \cdot 6H_2O$	4.80×10^{-8}
$CuCl_2 \cdot 2H_2O$	3.40×10^{-8}

Table S1c. Medium components of Vitamin Solution

Name of components	Concentration of components (g/ml)
Biotin	2.00×10^{-6}
Folic acid	2.00×10^{-6}
Thiamine-HCl	5.00×10^{-6}
D-calcium pantothenate	5.00×10^{-6}
cyanocobalamin	1.00×10^{-5}
riboflavin	5.00×10^{-6}
niacin	1.00×10^{-5}
Pyridoxal HCl	5.00×10^{-6}
p-aminobenzoic acid	5.00×10^{-6}
