# Supplementary material for: 2′-Deoxyuridine-promoted infection in Pyricularia oryzae is counteracted by bacterial thymidine phosphorylase
Source: Appl Environ Microbiol. 2026 Jul 13;92(8):e00727-26. doi: 10.1128/aem.00727-26 (PMC13488324; doi:10.1128/aem.00727-26)
Supplement: Supplementary file 7 [file aem.00727-26-s0007.pdf]

Table S2. DeoA-like proteins used for phylogenetic analysis

Cluster Composition	Cluster Ancestor	Representative sequence	Max Score	Total Score	Query Cover	E value	Per. ident	Acc. Len	Accession
33 member(s) 9 organism(s)	enterobacteria	thymidine phosphorylase [Leclercia sp. 29361]	868	868	100%	0	96.82	440	WP_166181548.1
10 member(s) 10 organism(s)	enterobacteria	thymidine phosphorylase [Klebsiella pneumoniae]	852	852	100%	0	95	438	WP_301688413.1
1 member(s) 1 organism(s)	enterobacteria	thymidine phosphorylase [Citrobacter freundii]	848	848	98%	0	95.84	560	QNC75940.1
5 member(s) 2 organism(s)	enterobacteria	Deoxyribose-phosphate aldolase [Salmonella enterica subsp. enterica serovar Stanley]	843	843	100%	0	95	723	VGM89610.1
14 member(s) 3 organism(s)	E. coli	thymidine phosphorylase [Escherichia coli]	838	838	100%	0	94.13	443	WP_139505100.1
1 member(s) 1 organism(s)	E. coli	thymidine phosphorylase [Escherichia coli]	838	838	100%	0	95.24	441	HIL7705332.1
3663 member(s) 427 organism(s)	bacteria	thymidine phosphorylase, partial [Klebsiella pneumoniae]	832	832	96%	0	95.76	425	WP_339353502.1
1 member(s) 1 organism(s)	enterobacteria	thymidine phosphorylase [Cedecea davisae DSM 4568]	825	825	100%	0	92.95	492	EPF20704.1
1 member(s) 1 organism(s)	enterobacteria	thymidine phosphorylase [Enterobacteriaceae bacterium ESL0689]	802	802	100%	0	88.86	440	MDF7679190.1
2016 member(s) 215 organism(s)	g-proteobacteria	thymidine phosphorylase, partial [Escherichia coli]	798	798	92%	0	96.29	404	WP_250384403.1
177 member(s) 15 organism(s)	enterobacteria	thymidine phosphorylase [Cronobacter sakazakii]	796	796	100%	0	88.86	440	WP_014729650.1
40 member(s) 19 organism(s)	root	thymidine phosphorylase, partial [Salmonella enterica]	791	791	94%	0	94.7	415	WP_200605900.1
13 member(s) 6 organism(s)	enterobacteria	thymidine phosphorylase [Edwardsiella hoshinae]	788	788	100%	0	85.91	440	WP_024524590.1
5 member(s) 3 organism(s)	enterobacteria	thymidine phosphorylase [Edwardsiella tarda]	786	786	100%	0	85.45	440	WP_416709845.1
1 member(s) 1 organism(s)	enterobacteria	thymidine phosphorylase [Entomohabitans teleogrylli]	782	782	100%	0	86.1	440	WP_058912140.1
1 member(s) 1 organism(s)	enterobacteria	thymidine phosphorylase [Edaphovirga cremea]	780	780	100%	0	85.23	440	WP_114192641.1
68 member(s) 26 organism(s)	enterobacteria	thymidine phosphorylase [Yersinia intermedia]	778	778	100%	0	85.68	440	WP_005191806.1
31 member(s) 10 organism(s)	enterobacteria	thymidine phosphorylase [Hafnia alvei]	777	777	100%	0	85.23	440	WP_367298519.1
2 member(s) 2 organism(s)	enterobacteria	thymidine phosphorylase [Obesumbacterium proteus]	777	777	100%	0	85.23	440	WP_046459867.1
346 member(s) 65 organism(s)	enterobacteria	thymidine phosphorylase [Yersinia similis]	777	777	100%	0	85.23	440	WP_145501067.1
1 member(s) 1 organism(s)	enterobacteria	thymidine phosphorylase [Intestinirhabdus alba]	774	774	99%	0	88.36	442	WP_155106711.1
1 member(s) 1 organism(s)	enterobacteria	thymidine phosphorylase [Erwinia sp. V71]	771	771	100%	0	84.32	440	WP_437612922.1
27 member(s) 17 organism(s)	g-proteobacteria	MULTISPECIES: thymidine phosphorylase [unclassified Erwinia]	766	766	100%	0	83.83	439	WP_339456953.1
1 member(s) 1 organism(s)	enterobacteria	MULTISPECIES: thymidine phosphorylase [Winslowiella]	764	764	100%	0	83.41	440	WP_313855547.1
2 member(s) 2 organism(s)	enterobacteria	thymidine phosphorylase [Shimwellia blattae]	761	761	100%	0	86.82	440	WP_002444034.1
111 member(s) 25 organism(s)	proteobacteria	thymidine phosphorylase [Providencia rustigianii]	761	761	100%	0	82.27	440	WP_112836141.1
1 member(s) 1 organism(s)	enterobacteria	thymidine phosphorylase [Enterobacterales bacterium CwR94]	761	761	100%	0	83.18	440	PKH22580.1
18 member(s) 14 organism(s)	enterobacteria	thymidine phosphorylase [Mixta hanseatica]	757	757	100%	0	82.27	440	WP_249893371.1
4 member(s) 4 organism(s)	enterobacteria	thymidine phosphorylase [Winslowiella arboricola]	754	754	100%	0	82.69	440	WP_267141345.1
48 member(s) 24 organism(s)	enterobacteria	MULTISPECIES: thymidine phosphorylase [unclassified Rahnella]	753	753	99%	0	84.93	462	WP_205965057.1
13 member(s) 4 organism(s)	enterobacteria	thymidine phosphorylase [Erwinia sorbitola]	750	750	100%	0	83.14	439	WP_154752754.1
2 member(s) 2 organism(s)	enterobacteria	thymidine phosphorylase [Salmonella enterica subsp. enterica serovar Typhimurium]	749	749	100%	0	87.05	403	ONF78716.1
7 member(s) 2 organism(s)	enterobacteria	thymidine phosphorylase [Gibbsiella quercinecans]	746	746	100%	0	81.82	440	WP_279026895.1
1 member(s) 1 organism(s)	enterobacteria	MULTISPECIES: thymidine phosphorylase [unclassified Erwinia]	746	746	100%	0	81.14	440	WP_056236382.1
1 member(s) 1 organism(s)	enterobacteria	thymidine phosphorylase [Paramixta manurensis]	745	745	100%	0	81.82	440	WP_173632795.1
8 member(s) 6 organism(s)	enterobacteria	thymidine phosphorylase [Erwinia billingiae]	744	744	100%	0	82	440	WP_346595532.1
5 member(s) 1 organism(s)	enterobacteria	thymidine phosphorylase [Moellerella wisconsensis]	743	743	100%	0	80.91	440	WP_241542006.1
4 member(s) 4 organism(s)	enterobacteria	thymidine phosphorylase [Chimaeribacter californicus]	742	742	99%	0	83.56	440	WP_101815027.1
3 member(s) 3 organism(s)	enterobacteria	thymidine phosphorylase [Serratia sp. DD3]	742	742	100%	0	81.14	440	WP_023489422.1
156 member(s) 36 organism(s)	proteobacteria	thymidine phosphorylase [Brenneria roseae]	742	742	100%	0	82.46	442	WP_109054646.1
70 member(s) 10 organism(s)	enterobacteria	MULTISPECIES: thymidine phosphorylase [Pantoea]	741	741	100%	0	82.73	442	WP_078829053.1
13 member(s) 7 organism(s)	enterobacteria	thymidine phosphorylase [Brenneria alni]	741	741	100%	0	82.23	443	WP_121575662.1
115 member(s) 23 organism(s)	enterobacteria	thymidine phosphorylase [Mixta mediterraneensis]	740	740	100%	0	82.95	442	WP_193406422.1
1 member(s) 1 organism(s)	enterobacteria	thymidine phosphorylase [Enterobacteriaceae bacterium 4M9]	740	740	100%	0	84.74	440	NDJ59018.1
142 member(s) 33 organism(s)	enterobacteria	thymidine phosphorylase, partial [Serratia bockelmannii]	740	740	95%	0	85.68	419	WP_380834107.1
19 member(s) 16 organism(s)	enterobacteria	thymidine phosphorylase [Pantoea sp. BAV 3049]	739	739	99%	0	81.51	440	WP_158781936.1
109 member(s) 14 organism(s)	enterobacteria	thymidine phosphorylase [Proteus mirabilis]	739	739	100%	0	79.77	440	WP_196748540.1
8 member(s) 8 organism(s)	enterobacteria	thymidine phosphorylase [Rouxiella sp. S1S-2]	739	739	99%	0	80.82	445	WP_152200886.1
1 member(s) 1 organism(s)	enterobacteria	thymidine phosphorylase [[Pantoea] beijingensis]	738	738	100%	0	80.23	440	WP_128178987.1
3 member(s) 3 organism(s)	root	thymidine phosphorylase [Pantoea deleyi]	738	738	100%	0	82.46	442	WP_375717330.1
1 member(s) 1 organism(s)	enterobacteria	thymidine phosphorylase [Enterobacillus tribolii]	737	737	100%	0	85.23	440	WP_115458740.1
1 member(s) 1 organism(s)	enterobacteria	thymidine phosphorylase [Rouxiella sp. Mn2063]	736	736	99%	0	82.42	445	WP_422792051.1
73 member(s) 23 organism(s)	enterobacteria	thymidine phosphorylase [Pantoea sp. OVA07A]	736	736	100%	0	82.05	442	WP_223566078.1
106 member(s) 7 organism(s)	enterobacteria	MULTISPECIES: thymidine phosphorylase [Morganella]	736	736	100%	0	80.91	440	WP_036417933.1
110 member(s) 61 organism(s)	bacteria	thymidine phosphorylase [Pantoea wallisii]	734	734	100%	0	82.73	440	WP_128599947.1
7 member(s) 3 organism(s)	enterobacteria	thymidine phosphorylase [Pantoea ananatis]	734	734	100%	0	81.67	444	WP_028723511.1
1 member(s) 1 organism(s)	enterobacteria	MULTISPECIES: thymidine phosphorylase [unclassified Pantoea]	734	734	100%	0	80.41	440	WP_147195007.1
5 member(s) 4 organism(s)	enterobacteria	thymidine phosphorylase [Mangrovibacter phragmitis]	731	731	100%	0	80.45	440	WP_064598666.1
4 member(s) 3 organism(s)	enterobacteria	MULTISPECIES: thymidine phosphorylase [Brenneria]	730	730	100%	0	80.18	443	WP_009114425.1
9 member(s) 7 organism(s)	enterobacteria	thymidine phosphorylase [Pantoea sp. SIMBA_079]	729	729	100%	0	79.77	440	WP_304235332.1
72 member(s) 16 organism(s)	enterobacteria	thymidine phosphorylase [Dickeya sp. NCPPB 3274]	729	729	99%	0	79.68	458	WP_050569932.1
4 member(s) 1 organism(s)	enterobacteria	thymidine phosphorylase [Serratia symbiotica]	729	729	100%	0	80.41	440	WP_006709112.1
34 member(s) 15 organism(s)	enterobacteria	thymidine phosphorylase [Candidatus Pantoea deserta]	728	728	100%	0	81.55	441	WP_123801556.1
1 member(s) 1 organism(s)	enterobacteria	Thymidine phosphorylase [Cronobacter sakazakii 680]	727	727	92%	0	87.96	407	CCK12768.1
2 member(s) 2 organism(s)	enterobacteria	thymidine phosphorylase [Tenebrionicola larvae]	724	724	100%	0	83.6	440	WP_249031958.1
1 member(s) 1 organism(s)	enterobacteria	thymidine phosphorylase [Izhakiella australiensis]	721	721	99%	0	79.68	439	WP_078002818.1
47 member(s) 27 organism(s)	enterobacteria	thymidine phosphorylase [Photorhabdus khanii]	721	721	100%	0	79.09	440	WP_152963511.1
1 member(s) 1 organism(s)	enterobacteria	thymidine phosphorylase [Musicola paradisiaca]	719	719	100%	0	78.82	444	WP_015854994.1
45 member(s) 11 organism(s)	enterobacteria	thymidine phosphorylase [Xenorhabdus lircayensis]	718	718	100%	0	78.7	446	WP_198689881.1
1 member(s) 1 organism(s)	enterobacteria	thymidine phosphorylase [Xenorhabdus bovienii]	718	718	99%	0	79.41	444	WP_434459933.1
6 member(s) 5 organism(s)	enterobacteria	thymidine phosphorylase [Brenneria uluponensis]	717	717	100%	0	77.68	447	WP_315708451.1
3 member(s) 2 organism(s)	enterobacteria	thymidine phosphorylase [Erwinia mallotivora]	716	716	99%	0	78.77	440	WP_261644293.1
6 member(s) 2 organism(s)	enterobacteria	thymidine phosphorylase [Duffyella gerundensis]	716	716	100%	0	77.73	440	WP_067427803.1
1 member(s) 1 organism(s)	enterobacteria	thymidine phosphorylase [Affinibrenneria salicis]	714	714	99%	0	77.85	443	WP_150433697.1
1 member(s) 1 organism(s)	enterobacteria	thymidine phosphorylase [Erwinia sp. 9145]	714	714	99%	0	79.91	440	WP_034912122.1
1 member(s) 1 organism(s)	enterobacteria	thymidine phosphorylase [Pantoea sp. 1.19]	713	713	99%	0	79.68	440	WP_075182659.1
53 member(s) 26 organism(s)	enterobacteria	thymidine phosphorylase, partial [Salmonella enterica]	712	712	82%	0	95.57	362	WP_139779668.1
8 member(s) 8 organism(s)	enterobacteria	thymidine phosphorylase [Pantoea sp. App145]	711	711	100%	0	81.36	440	WP_416411699.1
1 member(s) 1 organism(s)	enterobacteria	thymidine phosphorylase [Erwinia sp. HR93]	710	710	99%	0	80.14	443	WP_322837274.1
2 member(s) 2 organism(s)	enterobacteria	thymidine phosphorylase [Xenorhabdus mauleonii]	708	708	100%	0	77.3	446	WP_092513065.1
4 member(s) 3 organism(s)	enterobacteria	thymidine phosphorylase [Jejuibacter calystegiae]	707	707	99%	0	81.51	438	WP_462584806.1
8 member(s) 4 organism(s)	enterobacteria	thymidine phosphorylase [Salmonella enterica subsp. enterica serovar Bahrenfeld]	706	706	100%	0	77.05	453	HGB1822399.1
2 member(s) 2 organism(s)	enterobacteria	MULTISPECIES: thymidine phosphorylase [Pantoea]	705	705	99%	0	80.37	440	WP_322541487.1
1 member(s) 1 organism(s)	enterobacteria	MULTISPECIES: thymidine phosphorylase [unclassified Sodalis (in: enterobacteria)]	702	702	99%	0	77.4	442	WP_413735363.1
5 member(s) 3 organism(s)	enterobacteria	thymidine phosphorylase [Xenorhabdus doucetiae]	701	701	99%	0	75.94	455	WP_323857093.1
2 member(s) 2 organism(s)	enterobacteria	thymidine phosphorylase [Xenorhabdus sp. Sc-CR9]	701	701	100%	0	76.85	446	WP_237385364.1
2 member(s) 1 organism(s)	enterobacteria	thymidine phosphorylase [Erwinia psidii]	699	699	99%	0	77.63	440	WP_124231998.1
1 member(s) 1 organism(s)	enterobacteria	thymidine phosphorylase [Acerihabitans sp. TG2]	699	699	100%	0	76.77	442	WP_323480140.1
44 member(s) 13 organism(s)	g-proteobacteria	thymidine phosphorylase, partial [Shigella sonnei]	699	699	80%	0	96.58	351	WP_052984581.1
3 member(s) 3 organism(s)	enterobacteria	thymidine phosphorylase [Pantoea sp.]	697	697	100%	0	79.55	440	WP_312836970.1
2 member(s) 2 organism(s)	enterobacteria	thymidine phosphorylase [Xenorhabdus thailandensis]	695	695	99%	0	75.5	455	WP_340614462.1
12 member(s) 10 organism(s)	enterobacteria	MULTISPECIES: thymidine phosphorylase [Xenorhabdus]	694	694	99%	0	75.22	458	WP_047766697.1
5 member(s) 1 organism(s)	enterobacteria	thymidine phosphorylase [Xenorhabdus szentirmaii]	694	694	100%	0	75.68	445	WP_348991491.1
1 member(s) 1 organism(s)	enterobacteria	thymidine phosphorylase, partial [Gibbsiella quercinecans]	693	693	93%	0	81.51	411	WP_438596871.1
1 member(s) 1 organism(s)	enterobacteria	Thymidine phosphorylase [Klebsiella pneumoniae]	693	693	79%	0	96.84	400	CDL57910.1
1 member(s) 1 organism(s)	enterobacteria	thymidine phosphorylase [Xenorhabdus hominickii]	692	692	100%	0	75.82	455	WP_069316950.1
1 member(s) 1 organism(s)	enterobacteria	thymidine phosphorylase [Enterobacteriaceae bacterium]	692	692	99%	0	76.3	445	MDR0218429.1
8 member(s) 5 organism(s)	enterobacteria	thymidine phosphorylase [Xenorhabdus cabanillasii]	691	691	97%	0	76.85	465	WP_084766472.1
1 member(s) 1 organism(s)	enterobacteria	thymidine phosphorylase [Xenorhabdus kozodoii]	690	690	100%	0	75.39	452	WP_099142954.1
2 member(s) 2 organism(s)	enterobacteria	thymidine phosphorylase [Pantoea sp. A4]	687	687	100%	0	76.54	440	WP_017347649.1
