# Supplementary material for: 2′-Deoxyuridine-promoted infection in Pyricularia oryzae is counteracted by bacterial thymidine phosphorylase
Source: Appl Environ Microbiol. 2026 Jul 13;92(8):e00727-26. doi: 10.1128/aem.00727-26 (PMC13488324; doi:10.1128/aem.00727-26)
Supplement: Supplementary file 8 [file aem.00727-26-s0008.pdf]

Table S3. Primers used in this study.

Primer name	Sequence
Enterobacter_deoA_pET30_NotI_R	TGCTCGAGTGCGGCCCACCATAATAAATGCACGTTTCA
Enterobacter_deoA_pET30_EV_F	AAGGCCATGGCTGATGTGTTTCTCGCACAAGAAATTAT
1541r_16S	AAGGAGGTGATCCAGCCG
10f_16S	AGTTTGATCCTGGCTCAG
pPN94-linkerGFP-backbone-F	GGTGGTGGTGGTTCGATGGTGAGCAAGGGCGAGG
pPN94-Sp6-backbone-R	CGTCGAGTTCTATAGTGTCACCT
pPN94-Insert1-Sp6-pGAS2-F	CTATAGAACTCGACGCCTTGCAAATAGAGCTAGG
pPN94-Insert1-pGAS2-deoA-R	GTGCGAGAAACATTTTGGCGGTTTGAAATT
pPN94-Insert2-pGAS2-deoA-F	CGCCAAAATGTTTCTCGCACAAGAAATTATTCG
pPN94-Insert2-deoA-GFP-R	CATCGAACCACCACCACCTTCGGTGATACGGCGAT
